# Supplementary figures and images for: Establishment of a 12-gene expression signature to predict colon cancer prognosis
Source: PeerJ. 2018 Jun 14;6:e4942. doi: 10.7717/peerj.4942 (PMC6004299; doi:10.7717/peerj.4942)

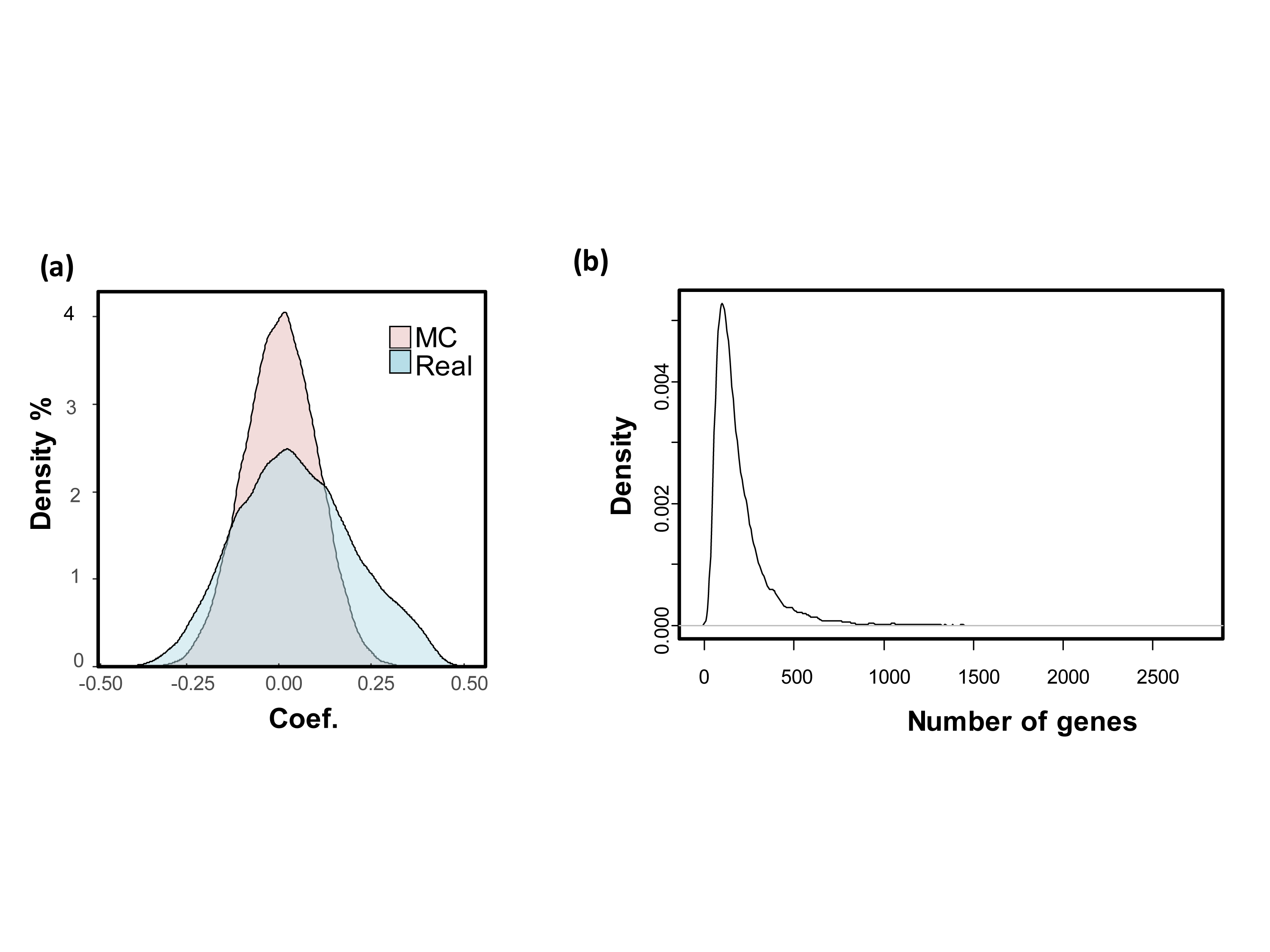

Supplement: Figure S1 — (A) Light green: correlation coefficient distributions between gene expression and prognosis categories for all the genes. There were 1,510 genes showed coefficient magnitude greater than 0.3; the light red distribution is one Monte-Carlo trial where the association of the gene expression and the prognostic category were randomized. (B) The frequency distribution of number of genes with absolute coefficient greater than 0.3 in the 10,000 Monte-Carlo trials. [file peerj-06-4942-s001.png]

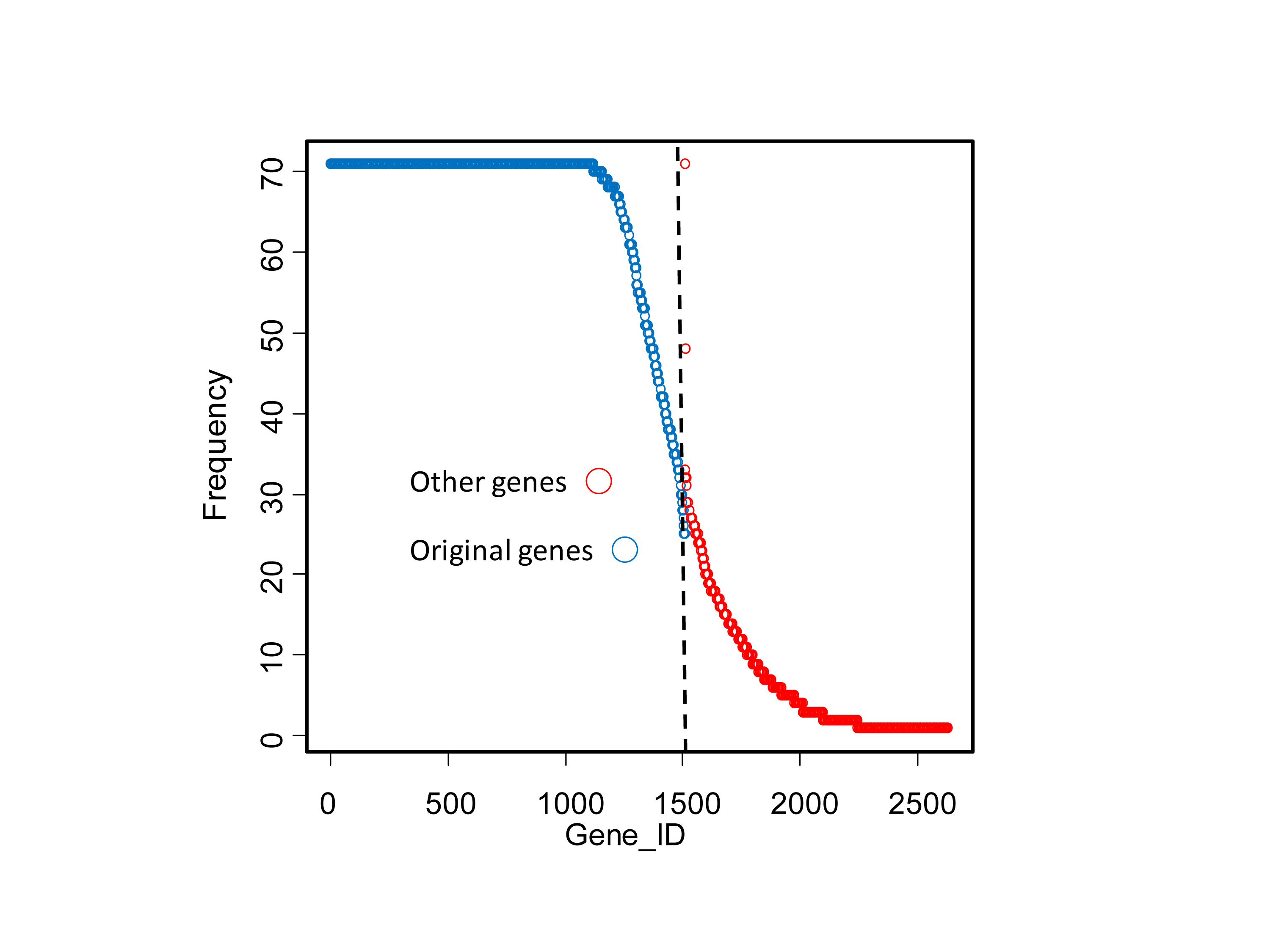

Supplement: Figure S2 — The average number of reporter genes is 1,519 ± 154 in one signature. [file peerj-06-4942-s002.png]
