# Supplementary material for: Establishment of a 12-gene expression signature to predict colon cancer prognosis
Source: PeerJ. 2018 Jun 14;6:e4942. doi: 10.7717/peerj.4942 (PMC6004299; doi:10.7717/peerj.4942)
Supplement: Table S3 [file peerj-06-4942-s005.docx]

| **Immune system related genes** | | **Association with cancer prognosis or outcomes** |
| --- | --- | --- |
| NCKIPSD | Innate immune system and regulation of actin dynamics for phagocytic cup formation. | NCKIPSD down-regulation inhibits drug uptake and tumor sensitivity to chemotherapy, resulting in high risk of breast cancer recurrence within 5 years(You et al., 2017). |
| TREML2 | Innate inflammatory responses; microglia activation; genetic risk factor for Alzheimer’s disease (AD). | None |
| TREML3 | Innate inflammatory responses; It has distinct structural and functional properties as TREM1 and 2. | None |
| PADI4 | Granulocyte and macrophage development; diseases associated with PADI4 include Rheumatoid Arthritis and Palindromic Rheumatism. | Cell free cirlation PADI4 mRNA may serve as a potential marker for NSCLC diagnosis (30). PADI4 has genetic susceptibility to gastric carcinoma and upregulates CXCR2, KRT14 and TNF-α expression levels(Zheng et al., 2016). |
| PTPRN | Autoantigen and autoimmunity in diabetes mellitus. | Hypermethylation in PTPRN was associated with longer progression-free survival in ovarian cancer (Bauerschlag et al., 2011). |
| PGLYRP1 | A pattern receptor that binds to murein peptidoglycans of Gram-positive bacteria; involved in Innate immune system and defensins. | PGLYRP1 (Tag7) interacts with Hsp70 to induces cytotoxic activity in tumor Cells via TNFR1 receptor (Yashin et al., 2015). |
| C5orf53 | Enhances IgA secretion from B-cells stimulated via CD40; post-meal increment in C5orf53(IGIP1) level is inversely associated with metabolic syndrome in patients with type 2 diabetes. | None |
| **Neuron function related genes** | |  |
| FAM171B | Expressed in mouse brain. Supposed to be potentially linked to a neurodegenerative disease. | FAM171B was up-regulated in gemcitabine-resistant pancreatic cancer cell line (Zhou et al., 2015). |
| NKAIN4 | A protein with similarity to Drosophila Nkain and interacts with the beta subunit of Na, K-ATPase. May be critical for neuronal function. | None |
| **Sporadic function** | |  |
| NOG | A secreted polypeptide, which binds and inactivates members of the TGF-β superfamily signaling proteins, such as BMP4, creating morphogenic gradients. | High expressing of gene signature including NOG showed a strong trend for a worse prognosis of patients with lung adenocarcinomas (Rajski et al., 2015). |
| VIP | Myocardial contractility; antimicrobial activity | Enhanced VIP signaling in human prostate cancer (Fernandez-Martinez et al., 2012). Enhanced VIP secretion is associated with advanced tumor stage in colorectal carcinoma (Hirayasu et al., 2002). |
| RIMKLB | Alanine, aspartate and glutamate metabolism | RIMKLB up-regulation is associated with radio-resistance in nasopharyngeal carcinomas (Li et al., 2016). |
